# Structural plasticity of the axon initial segment in rat hippocampal granule cells following high frequency stimulation and LTP induction

**DOI:** 10.3389/fnana.2023.1125623

**Published:** 2023-04-05

**Authors:** Tassilo Jungenitz, Alexander Bird, Maren Engelhardt, Peter Jedlicka, Stephan W. Schwarzacher, Thomas Deller

**Affiliations:** ^1^Institute of Clinical Neuroanatomy, Goethe University Frankfurt, Frankfurt am Main, Germany; ^2^Interdisciplinary Centre for 3Rs in Animal Research, Justus Liebig University Giessen, Giessen, Germany; ^3^Institute of Anatomy and Cell Biology, Johannes Kepler University Linz, Linz, Austria

**Keywords:** dentate gyrus, neuronal plasticity, synaptopodin, cisternal organelle, entorhinal cortex, perforant path, axon initial segment

## Abstract

The axon initial segment (AIS) is the site of action potential initiation and important for the integration of synaptic input. Length and localization of the AIS are dynamic, modulated by afferent activity and contribute to the homeostatic control of neuronal excitability. Synaptopodin is a plasticity-related protein expressed by the majority of telencephalic neurons. It is required for the formation of cisternal organelles within the AIS and an excellent marker to identify these enigmatic organelles at the light microscopic level. Here we applied 2 h of high frequency stimulation of the medial perforant path in rats *in vivo* to induce a strong long-term potentiation of dentate gyrus granule cells. Immunolabeling for βIV-spectrin and synaptopodin were performed to study structural changes of the AIS and its cisternal organelles. Three-dimensional analysis of the AIS revealed a shortening of the AIS and a corresponding reduction of the number of synaptopodin clusters. These data demonstrate a rapid structural plasticity of the AIS and its cisternal organelles to strong stimulation, indicating a homeostatic response of the entire AIS compartment.

## Introduction

The axon initial segment (AIS) is an unmyelinated electrogenic domain in the proximal axon, characterized by a high concentration of voltage-gated ion channels. Its location and channel architecture are the structural and molecular basis for the lowest threshold for action potentials within a neuron and, thus, it integrates synaptic input and determines neuronal output (Palay et al., 1968; Kole et al., 2008; Clark et al., 2009; Bender and Trussell, 2012; Kole and Stuart, 2012). The AIS contains ring-forming actin-filaments arranged in 190 nm repeats. These structures are organized by Ankyrin-G (AnkG), which interacts with spectrin tetramers (αII-/βIV-spectrin) and a number of adaptor proteins (Xu et al., 2013; Zhong et al., 2014; Leterrier et al., 2015; Huang et al., 2017). Location, length, diameter and channel composition of the AIS are dynamic, modulating the initiation of action potentials and thereby contributing to the homeostatic control of firing probabilities (reviewed in Petersen et al., 2017; Jamann et al., 2018).

Synaptopodin is an actin-modulating and plasticity-related protein found in kidney podocytes and neurons (Mundel et al., 1997). It is required for the formation of the cisternal organelle (CO) in the AIS and the spine apparatus in dendritic spines (Deller et al., 2000, 2003, 2007; Bas Orth et al., 2007). Mice lacking synaptopodin exhibit deficits in Hebbian (Jedlicka and Deller, 2017) as well as homeostatic (Vlachos et al., 2013) forms of synaptic plasticity. The CO of the AIS consists of stacks of smooth endoplasmic reticulum and dense plates (Peters et al., 1968; Kosaka, 1980; Spacek, 1985; Benedeczky et al., 1994). In analogy to the spine apparatus, it has been suggested that the CO plays a role in intra-axonal Ca^2+^ homeostasis (Bas Orth et al., 2007; Schafer et al., 2009; Sánchez-Ponce et al., 2011). Its presence and distribution within the AIS have recently been studied during cortical development, suggesting that changes of the CO could be a feature of structural AIS plasticity (Schlüter et al., 2017, 2019).

In this study, we investigated how the AIS and the CO of dentate granule cells (GCs) react to strong afferent stimulation. Using 2 h high-frequency stimulation (HFS) of the medial perforant path (MPP), robust functional and structural changes of GCs are induced: In the stimulated middle molecular layer, homosynaptic LTP and an increase in dendritic spine sizes are seen following stimulation (Bliss and Lomo, 1973; Jungenitz et al., 2014, 2018). In the non-stimulated outer molecular layer, heterosynaptic LTD (Fukazawa et al., 2003; Abraham et al., 2007; Beining et al., 2017a; Jungenitz et al., 2018) and a decrease in dendritic spine sizes have been reported (Beining et al., 2017a; Jungenitz et al., 2018). We now asked whether this stimulation protocol, which induces remarkable dendritic changes, also affects the AIS and the CO of GCs. βIV-spectrin was used as a marker to identify the AIS in GCs and revealed a rapid shortening of the AIS in response to stimulation. Synaptopodin immunolabeling demonstrated that this decrease in length was accompanied by a decrease in synaptopodin clusters, indicative for a reduction of COs. Together, our data demonstrate that the AIS and the CO of GCs rapidly respond to MPP stimulation and thus participate in the plastic response of GCs to changes in their afferent drive.

## Materials and methods

### Animals

Adult male Sprague-Dawley rats (8–13 weeks, 220–450 g; Charles River, Sulzfeld, Germany) were housed under standard laboratory conditions. All animal experiments were reviewed by the local ethics committee (Regierungspräsidium Darmstadt, Dezernat V 54; permit number F6/18) and performed in agreement with the German laws (TSG) for the use of animals in research.

### Adeno-associated virus production

Pseudotyped adeno-associated viral (AAV) particles were generated using a helper virus free packaging method. HEK293T cells were co-transfected with pDP1rs (Plasmid Factory), pDG (Plasmid Factory) and AAV2-hSyn1-GFP vector-plasmid (Shevtsova et al., 2005) (6:4:1) by Calcium-Phosphate precipitation (protocol adapted from Grimm et al., 1998; Grimm, 2002). The transfected cells were collected 48 h after transfection. Cells were washed (2 times) by centrifugation at 1,500 × *g* for 5 min and resuspended in PBS. The viral particles inside the cells were set free by multiple freeze thaw cycles (four times). The supernatant was collected and washed by centrifugation at 3,200 × *g* for 10 min. The AAV containing supernatant was aliquoted and stored at −80°C.

### Intra-hippocampal viral *in vivo* injection

The following surgical procedures were performed under deep Medetomidin (Domitor; Pfizer, New York City, NY, USA), Midazolam (Dormicum; Roche, Basel, Switzerland) and Fentanyl (Janssen Pharmaceutica, Beerese, Belgium) anesthesia (150 μg Medetomidin, 2 mg Midazolam, 5 μg Fentanyl per kg body weight i.m. initially and additional injections as needed). Animals were placed in a Kopf stereotaxic device (Kopf Instruments, Tujunga, CA, USA). Two small holes (1.5-2.0 mm diameter) were drilled in the skull at −3.8 mm from Bregma and 2.2 mm laterally at both hemispheres. A NanoFil syringe (World Precision Instruments, Inc., Sarasota, FL, USA) with a 35 gauge beveled needle (NF35BV-2; World Precision Instruments) was used to slowly inject 0.75 μl of the viral solution at 3.2 mm and 3.7 mm below the brain surface into the dentate gyrus (DG; both hemispheres).

### Perforant path stimulation *in vivo*

All surgical procedures were performed under deep urethane anesthesia (1.25 g/kg body weight s. c. initially and additional injections as needed; 250 mg urethane/ml 0.9% saline). Surgery and stimulation procedures were performed as previously described (Schwarzacher et al., 2006; Jedlicka et al., 2009). In short, animals were placed in a Kopf stereotaxic device (Kopf instruments, Tujunga, CA, USA). Rectal temperature was maintained at 37.0 ± 0.5°C. Two small holes (1.5 – 2.0 mm diameter) were drilled in the skull and a bipolar stainless steel stimulating electrode (NE-200; Rhodes Medical, Woodland Hills, CA, USA) was placed in the angular bundle of the perforant path (coordinates from lambda: L: 4.5 mm; AP: +0.5 mm; V: −3.5 mm measured from the surface of the brain). Glass microelectrodes (1.5 mm outer diameter) were pulled on a Zeitz (München, Germany) electrode puller, filled with 0.9% saline, and placed in the dorsal blade of the granule cell layer (GCL; coordinates from bregma: L: 2.0 mm, AP: −3.5 mm, V: −3.5 mm). The vertical tip position was optimized under perforant path control stimulation using the characteristic shape of the evoked potentials.

High frequency stimulation (HFS) was used to maximally evoke population spikes and induce robust long term potentiation (LTP) as has been described in detail (Steward et al., 1998). HFS was applied for 2 h. One HFS train consisted of 8 pulses (500 μA, 0.1 ms pulse duration) of 400 Hz once per 10 s. A baseline fEPSP slope was calculated from the average of responses over the 10 min prior to the theta-burst stimulation (TBS). Baseline stimulus intensity was set to evoke a population spike of approximately 1 mV before LTP induction. The potentiation of the fEPSP slope was expressed as percentage change relative to baseline.

Rats were transcardially perfused with a fixative containing 4% paraformaldehyde in 0.1 M phosphate buffered saline (PBS), pH 7.4. Brains were removed and postfixed up to 18 h in 4% paraformaldehyde in 0.1 M PBS.

### Tissue preparation

Serial frontal sections of the hippocampus (50 μm) were cut with a vibratome, washed in 0.1 M TRIS buffered saline (TBS; AppliChem, Darmstadt, Germany), and stored at –20°C in cryoprotectant solution (30% ethylene glycol, 25% glycerin in 0.1 M PBS).

### Immunohistochemistry

Free-floating sections were washed in TBS, blocked with 5% bovine serum albumin (BSA; New England BioLabs, Ipswich, MA, USA) containing 0.1% Triton X-100 for 1 h at room temperature to reduce non-specific staining and incubated in primary antibody solution containing 2% BSA, 0.25% Triton X-100, 0.1% NaN_3_ in 0.1 M TBS for 48 h at room temperature. The following primary antibodies were used: anti-βIV-spectrin (rabbit, 1:500; selfmade, see Schlüter et al., 2017), anti-synaptopodin (rabbit, polyclonal, 1:1,000; Synaptic Systems, Göttingen, Germany) and anti-GFP488 (mouse, 1:500, fluorescence-labeled Alexa 488; Sigma-Aldrich). For immunofluorescence detection, sections were incubated with secondary fluorescence-labeled antibodies (1:1,000; Alexa 488, 568, 633; Vector Labs., Burlingame, CA, USA) for 24 h at room temperature.

### Image acquisition and analysis of axon initial segments

Image acquisition was performed on six animals in total and one frontal section of the dorsal DG per animal. Only sections showing an accumulation of synaptopodin in the stimulated ipsilateral middle molecular layer indicating successful LTP induction (Fukazawa et al., 2003) were used. For each section, six image stacks (three from the stimulated ipsilateral and three from the non-stimulated contralateral hemisphere) of the suprapyramidal GCL and subgranular zone (SGZ) of the DG were acquired using a confocal microscope (Nikon C2 plus) and a 60× oil immersion objective (N.A. 1.3; Nikon) with a 2× field zoom.

Image analysis was performed with Fiji (Schindelin et al., 2012). βIV-spectrin as an important structural protein of the AIS was utilized to identify segments. Only segments completely contained within the image stack and without showing obfuscating collisions with neighboring segments were selected and reconstructed in 3D using the SNT framework (Arshadi et al., 2021). Some granule cell somata and the initial part of axons were reconstructed based on the GFP-expression induced by the adeno-associated virus under the synapsin promotor (n_*contralateral*_ = 10, n_*ipsilateral*_ = 7) to obtain the distance between soma and AIS.

In order to identify synaptopodin clusters located in the AIS, the synaptopodin image channel of each image stack was filtered (median and mean filter), thresholded (Auto Local threshold—type MaxEntropy) and segments were scanned for synaptopodin clusters along their reconstructed trace. The largest cross-sectional area of each cluster was selected and assigned to the related segment.

Morphological measurements (e.g., position, AIS length, synaptopodin cluster number and area) were exported and analyzed.

### Compartmental modeling

Granule cell voltage simulations were carried out in Matlab (R2022b) and Neuron (v 7.8.1) using the Trees Toolbox (Cuntz et al., 2010) and T2N (Beining et al., 2017b) packages. 20 rat granule cell morphologies (5 reconstructed, 15 synthetic) and their fitted channel conductances were taken from Beining et al., 2017a. To determine the stimulation protocol for each morphology, constant currents of 100 ms duration were injected into the soma with amperages increasing in steps of 0.01 nA until the model produced an action potential. The morphology then had its original AIS decreased and increased in size by 18, 9, and 4%, with the total length of the axon and the AIS remaining constant, giving seven models for each original morphology. This reflected the change observed in our experiments and multiples, i.e., 2×, 4×, thereof. The somatic voltage was simulated for each different AIS size in response to the stimulation protocol determined for the original morphology.

The spike latency was measured as the time from the onset of current injection to the peak of the action potential. The action potential height was measured as the difference in voltage from the peak of the action potential to its value 10 ms before the peak. The afterhyperpolarisation depth was measured as the difference in voltage from 10 ms before the action potential peak to the minimum voltage attained in the 10 ms after the peak. The black lines in Figures 4C, D show averages over the 20 morphologies and the green shaded areas show the 90% confidence interval.

### Digital illustrations

**FIGURE 1:**
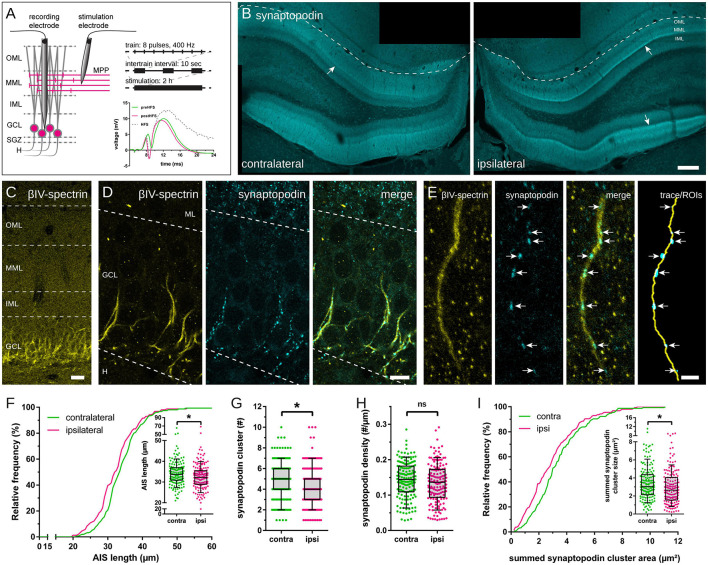
Axon initial segment (AIS) and synaptopodin in granule cells (GCs) following 2 h high frequency stimulation (HFS) of the perforant path. **(A)** HFS (eight pulses at 400 Hz, 10 sec intertrain interval) was applied to induce long-term potentiation (LTP) of the medial perforant path for 2 h. Local field potentials of GCs were recorded before (green), during (gray) and after HFS (red) to estimate LTP induction. **(B)** Accumulation of synaptopodin in the middle molecular layer (MML; indicated by arrows) of the stimulated, ipsilateral dentate gyrus (DG) confirmed successful LTP induction. **(C)** The scaffolding protein βIV-spectrin was used to localize AIS of GCs and **(D)** co-labeling of synaptopodin revealed synaptopodin clusters within the AIS, indicative of cisternal organelles (CO). **(E)** AIS were reconstructed in image stacks, colocalized synaptopodin clusters (indicated by arrows) were identified and their largest cross-sectional area was measured. **(F)** AIS located in the ipsilateral hemisphere revealed a significant shortening following 2 h HFS. **(G)** The absolute number of synaptopodin clusters inside individual AIS decreased significantly, although synaptopodin cluster density remained unchanged **(H)**. **(I)** The total (summed) area of synaptopodin clusters in individual segments was significantly reduced following 2 h HFS. Number of segments: **(F–I)** n_contralateral_ = 155, n_ipsilateral_ = 156. Box and whiskers represents 10–90 percentile. *p < 0.05, Mann–Whitney test. Scale bars: **(B)** 200 μm, **(C)** 25 μm, **(D)** 10 μm, **(E)** 2.5 μm. GCL, granule cell layer; H, hilus; IML, inner molecular layer; ML, molecular layer.

Confocal images were edited and stitched (Figure 1B) with Fiji (Schindelin et al., 2012). Figures were prepared with Adobe Photoshop and Adobe Illustrator (Adobe Inc., San Jose, CA, USA). Contrast and brightness were adjusted. To increase visibility of the axon shown in 3C, areas above the depicted axon were removed from the confocal image stack before generating the z projection. Apart from this, no additional image alteration was performed.

### Statistical analysis

Data management, statistical analysis, and visualization were done with Microsoft Excel (Microsoft, Redmond, WA, USA) and GraphPad Prism 6.07 (Graphpad Software, San Diego, CA, USA). Statistical comparisons were calculated using the Mann–Whitney test. The significance level was set at *p* < 0.05.

## Results

### High-frequency stimulation of the perforant path induces reduction of axon initial segment length and number of synaptopodin clusters

Two hours HFS of the medial perforant path (MPP) were performed using an established stimulation protocol (Figure 1A) to induce robust unilateral LTP in granule cells (GCs) of the ipsilateral DG, with the contralateral DG serving as non-stimulated control (Jungenitz et al., 2014, 2018; Beining et al., 2017a). This 2 h HFS resulted in an increased slope, population spike, expression of immediate early genes (e.g., Arc), and a structural remodeling of dendritic spines in GCs of the ipsilateral DG (Beining et al., 2017a; Jungenitz et al., 2018). Furthermore, 2 h HFS resulted in an accumulation of synaptopodin in the stimulated middle molecular layer (MML) of the ipsilateral DG (Figure 1B; c.f. Fukazawa et al., 2003).

Axon initial segments located in the GCL and the subgranular zone (SGZ) of the contralateral, non-stimulated and the ipsilateral, stimulated hemisphere were identified, imaged and reconstructed in three dimensions (Figures 1C–E). AIS located in the non-stimulated DG showed an average length of 34.14 ± 0.47 μm (mean ± SEM; *n* = 155; number of animals = 6; Figure 1F). In comparison, 2 h HFS led to a significant reduction of AIS located in the stimulated DG by 4.42% to 32.63 ± 0.51 μm (*n* = 156; number of animals = 6; Mann–Whitney test, *p* = 0.01).

**FIGURE 2:**
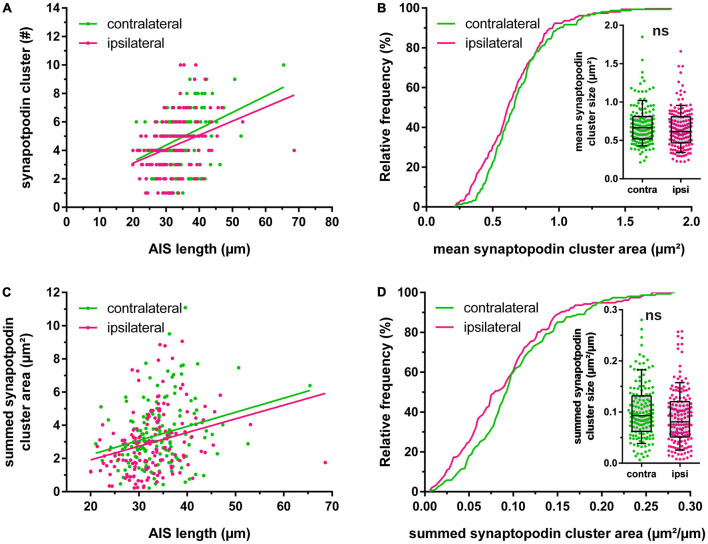
Homeostatic adaptation of synaptopodin. **(A)** Relationship between length of the axon initial segment (AIS) and absolute number of synaptopodin clusters. **(B)** The mean area of synaptopodin clusters was not significantly altered following 2 h high frequency stimulation. **(C,D)** The summed area of synaptopodin clusters was normalized to the AIS length and showed no significant changes between stimulated vs. non-stimulated granule cells. Number of segments: **(A–D)** n_contralateral_ = 155, n_ipsilateral_ = 156. Box and whiskers represent 10–90 percentile. ns > 0.05, Mann–Whitney test.

Synaptopodin clusters localized to reconstructed AIS were counted and their largest cross-sectional area was measured. The absolute number of synaptopodin clusters per AIS was 4.85 ± 0.15 clusters on the non-stimulated hemisphere, but 2 h HFS reduced this number significantly to 4.35 ± 0.16 clusters (Figure 1G; Mann–Whitney test, *p* = 0.01). Summation of individual synaptopodin cluster areas added up to an average of 3.44 ± 0.15 μm^2^ per AIS located in the non-stimulated hemisphere and was significantly decreased to 2.96 ± 0.15 μm^2^ following HFS (Figure 1I; Mann–Whitney test, *p* = 0.01). Of note, although the absolute number of synaptopodin clusters was found to be reduced after stimulation, neither the density of synaptopodin clusters (Figure 1H; non-stimulated: 0.143 ± 0.004 cluster/μm, stimulated: 0.134 ± 0.004 cluster/μm; Mann–Whitney test, *p* = 0.10), nor the mean size of synaptopodin clusters were found to be altered (Figure 2B; non-stimulated: 0.70 ± 0.02 μm^2^, stimulated: 0.65 ± 0.02 μm^2^; Mann–Whitney test, *p* = 0.11). Because of these observations, we normalized the number of synaptopodin clusters to the AIS length and found no significant difference between stimulated and non-stimulated GCs (Figure 2D; non-stimulated: 0.101 ± 0.004 μm^2^/μm, stimulated: 0.090 ± 0.004 μm^2^/μm; Mann–Whitney test, *p* = 0.05). Those findings strongly suggest that the density of COs in the AIS is fine-tuned to its length, i.e., the homeostatic response of the AIS to stimulation includes not only a length adaptation but also a homeostatic adaptation of the number of COs within the AIS.

**FIGURE 3:**
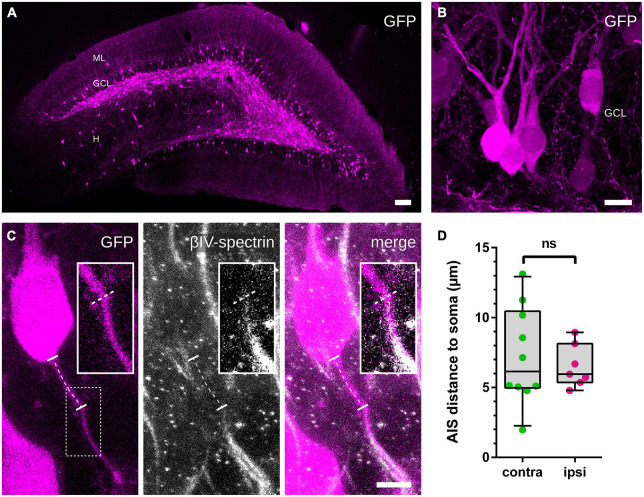
Distance between soma and AIS. **(A,B)** An AAV2-hSyn1-GFP was injected into the hilus 14 days prior to HFS and induced a robust GFP expression in GCs, which was used to identify GC somata and originating axons. **(C)** Colocalization with βIV-spectrin revealed the beginning of the AIS and the path of the axon between the soma and the AIS was traced (indicated by the dotted line). **(D)** Measured distances revealed no significant differences between the non-stimulated and the stimulated hemispheres. Number of axons: **(D)** n_contralateral_ = 10, n_ipsilateral_ = 7. Box and whiskers represents 10–90 percentile. ns > 0.05, Mann–Whitney test. Scale bars: **(A)** 100 μm, **(B)** 10 μm, **(C)** 5 μm. GCL, granule cell layer; H, hilus; ML, molecular layer.

The injection of an AAV2-hSyn1-GFP into the hilus 14 days prior to HFS resulted in a robust GFP expression (see Jungenitz et al., 2018) and was used to identify GFP-labeled GCs and their axons (Figures 3A, B). By tracing the path between the somatic origin of the axon and the start of its AIS (identified by colocalization with βIV-spectrin), we were able to measure the distance of the AIS from the soma (Figure 3C). Comparisons between the non-stimulated and the stimulated hemispheres showed no significant stimulation-dependent changes of the distance (Figure 3D; non-stimulated: 7.2 ± 1.1 μm (*n* = 10), stimulated: 6.5 ± 0.6 μm (*n* = 7); Mann–Whitney test, *p* > 0.99).

### Modeling predicts that reduced AIS size decreases intrinsic excitability

**FIGURE 4:**
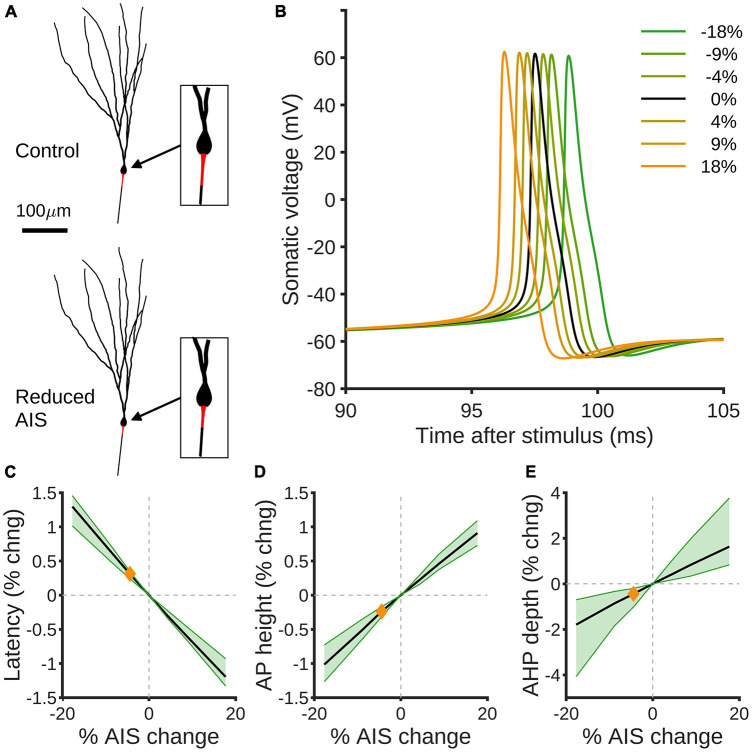
Compartmental modeling predicts effects of AIS size reduction on spiking properties of GCs. **(A)** Example rat granule cell morphology (Beining et al., 2017a) with control (top) and 18% reduced (bottom) AISs highlighted in red. **(B)** Simulated somatic voltage traces in response to somatic current injection. The black curve shows the control morphology, green curves show reduced AISs and orange curves increased AISs. **(C)** Percentage change in spike latency as a function of percentage change in AIS size. Black shows the mean effect, green the 90% confidence interval over different morphologies, and the orange diamond indicates the effect of the observed experimental reduction in AIS size by 4.42%. **(D,E)** Percentage changes in spike height **(D)** and after-hyper polarization depth **(E)** as a function of percentage change in AIS. Colors as in panel **(C)**.

We used compartmental modeling to isolate the impact of changing AIS size on granule cell excitability (Figure 4A). By altering the AIS size in a previously published granule cell model (Beining et al., 2017a), we found small but highly consistent effects on the spiking behavior in response to somatic current injection (Figure 4B). In particular, decreased AIS size implies that spike latency is increased (Figure 4C) and action potential size is decreased (Figure 4D), but that afterhyperpolarisation depth is also decreased (Figure 4E). The AIS contains relatively high densities of Kv3.3/4 and Kv7.2/3 voltage-dependent K-channels, SK1.3 calcium-dependent K-channels, and Nav1.6 voltage-dependent Na-channels compared to the remainder of the axon; if the densities of such channels in the AIS remain the same despite shrinkage, our modeling predicts a decrease in intrinsic excitability, as measured by latency and action potential size, in response to HFS.

## Discussion

**FIGURE 5:**
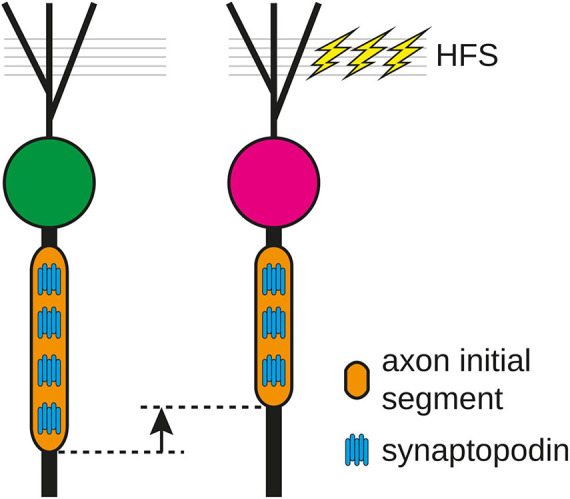
Following 2 h high frequency stimulation (HFS) of the medial perforant path, the length of the axon initial segment (AIS) and the number of synaptopodin clusters were reduced, indicating that the number of cisternal organelles in the AIS is fine-tuned to its length and can be modified as a homeostatic response of the AIS to stimulation.

In the present study, we provide evidence for a rapid structural remodeling of the AIS of rat GCs in response to 2 h HFS of the MPP *in vivo*. Following stimulation, the AIS of GCs were significantly shorter without changing their distance to the soma. This reduction in length went hand-in-hand with a reduction in the absolute number of COs and the CO density was maintained within the shortened AIS, demonstrating that the AIS modifies not only its length but also its organelle composition following HFS. In line with this, compartmental modeling predicted that the reduction of AIS prolongs spike latency potentially decreasing intrinsic excitability of GCs. Taken together, these adaptations of the AIS counteract the increased afferent drive and likely represent a rapid homeostatic response of the stimulated GC (Figure 5).

High-frequency stimulation of the medial perforant path leads to the induction of homo- and heterosynaptic plasticity at GC-synapses with opposing effects (Abraham et al., 1985, 2007; Jungenitz et al., 2018). Directly stimulated synapses in the MML exhibit LTP, associated with the structural enlargement of dendritic spines and an increase of F-actin, as well as synaptopodin. In contrast, adjacent non-stimulated synapses exhibited LTD in combination with a reduction in spine size (Fukazawa et al., 2003; Jungenitz et al., 2018), highlighting the role of heterosynaptic plasticity as a homeostatic mechanism for normalization of synaptic weight. The AIS is important for signal integration and initiation of action potentials. Its physical parameters such as length, location, diameter and channel architecture have been shown to undergo structural plasticity during normal development (Gutzmann et al., 2014; Le Bras et al., 2014; Kim et al., 2019) and as a response to changes in network state *in vitro* and *in vivo* (Grubb and Burrone, 2010; Kuba et al., 2010; Scott et al., 2014; Evans et al., 2015; Jamann et al., 2021; Wu et al., 2022). These types of structural remodeling at the AIS may have direct impact on cellular excitability (Grubb and Burrone, 2010; Kuba et al., 2010; Evans et al., 2015; Jamann et al., 2021). Consequently, the AIS is involved in homeostatic scaling of cellular excitability by influencing neuronal input/output parameters (reviewed in Wefelmeyer et al., 2016; Jamann et al., 2018). As outlined below, recent studies indicate both cell-type specific as well as distinct temporal dynamics for AIS plasticity.

Under physiological conditions, the AIS has been shown to undergo periods of structural plasticity during normal development. In the visual system, AIS length correlates with the opening and closure of critical periods of cortical and retinal development (Gutzmann et al., 2014; Schlüter et al., 2017, 2019), while in the mouse auditory brainstem and avian nucleus magnocellularis, the structure and spatial location of the AIS depend on the functional topographic location of neurons along the tonotopic axis (Kuba et al., 2010; Kim et al., 2019). Similarly, adult born hippocampal GCs undergo developmental AIS plasticity, with the most significant changes coinciding with periods of morphological and functional remodeling (Bolós et al., 2019).

Here, we report rapid AIS shortening after only 2 h of HFS and induction of LTP. Indeed, the prominent structural plasticity observed *ex vivo* and *in vivo* are changes in AIS length (elongation, shortening). In this context, different temporal windows for length remodeling have been reported. On one hand, AIS elongation, driven by a reduction in synaptic drive or complete blockage of network activity required days to weeks of exposure for example in the avian nucleus magnocellularis *in vivo* (Kuba et al., 2010), mouse primary visual cortex *in vivo* (Gutzmann et al., 2014; Schlüter et al., 2017), or mouse barrel cortex *in vivo* (Jamann et al., 2021). Whole-cell patch clamp recordings in these settings showed a significant increase in neuronal excitability after AIS elongation (Kuba et al., 2010; Jamann et al., 2021). On the other hand, AIS shortening only requires minutes to hours to occur after network states are altered. Strikingly, not only non-physiological conditions such as high extracellular potassium (Grubb and Burrone, 2010; Evans et al., 2015), but also network stimulations in a physiological context, resulted in rapid AIS shortening (Jamann et al., 2021). Likewise, a recent study reported an NMDA-receptor-dependent reduction in AIS length within half an hour after induction of chemical long-term depression (Fréal et al., 2022).

Our findings are well in line with these reported activity-dependent changes in both structure and function of the AIS. In fact, they highlight the role of synaptic drive on AIS architecture and subsequent cellular function. Previous work in hippocampal GCs *in vitro* showed that repetitive firing elicited by optogenetic stimulation for 3 h resulted in a significant reduction of AIS length and decrease in neuronal excitability (Evans et al., 2015). Here, we report rapid AIS shortening after only 2 h of high frequency stimulation *in vivo* and induction of LTP. Taken together, these data support the hypothesis that the AIS can undergo rapid and highly dynamic remodeling in a temporal frame that would allow for an interaction with other forms of activity-dependent plasticity.

We did not observe a change in the distance of the AIS to the soma following our stimulation protocol (Figure 3). In pathophysiological conditions such as chronic depolarization *in vitro*, introduced by either the elevation of extracellular potassium or photostimulation, the AIS undergoes a distal shift along the axon (Grubb and Burrone, 2010; Evans et al., 2013; Wefelmeyer et al., 2015, 2016). A similar effect was observed after selective M-channel and synaptic receptor blockage *in vitro* (Lezmy et al., 2017, 2020). In all these studies, AIS relocation was accompanied by a reduction of neuronal excitability. Of note, recent theoretical predictions (Kole and Brette, 2018; Goethals and Brette, 2020) as well as experimental evidence (Fékété et al., 2021) indicate that the opposite effect—an increase in neuronal excitability after a distal AIS shift—is just as likely, highlighting the fact that our understanding of the exact implication of structural plasticity on cellular function remains incomplete and certainly depends on the model parameters used.

Rapid shortening of AIS following HFS coincided with a significant decrease of synaptopodin clusters per AIS, but no alteration of synaptopodin cluster size (Figure 2). Various reports indicate that AIS function (Bender and Trussell, 2009; Hanemaaijer et al., 2020) and structural AIS plasticity (Schlüter et al., 2017) depend on changes in intraaxonal Ca^2+^ levels. Specifically, the activation of plasticity-type downstream signaling pathways including calcineurin have been implicated in AIS remodeling *in vitro* (Grubb and Burrone, 2010; Evans et al., 2013, 2015). Due to its strategic location within the AIS, the CO may serve as a intracellular Ca^2+^ source or sink (Benedeczky et al., 1994; Bas Orth et al., 2007). Synaptopodin is essential for the formation of the CO, as synaptopodin-KO mice lack the CO (Bas Orth et al., 2007). In visual cortex development, synaptopodin is increasingly expressed during neuronal maturation and is stabilizing the AIS (Schlüter et al., 2017). Strikingly, reduction in sensory input via sensory deprivation led to structural remodeling not only of the AIS, but also the CO in both primary visual cortex pyramidal neurons (Schlüter et al., 2017) as well as retinal ganglion cells (Schlüter et al., 2019). Considering that the reduction in AIS length and number of CO clusters likely results in reduced cellular excitability and decreased Ca^2+^ storage capacity, these changes may be interpreted as homeostatic responses of GCs after HFS aimed at counterbalancing the increase in synaptic drive (Figure 5). Together with the capability of dendritic spines for heterosynaptic structural plasticity, this work presents evidence for an additional homeostatic mechanism to maintain neurons within their physiological operating ranges.

## Data availability statement

The data will be made available by the corresponding author upon reasonable request.

## Ethics statement

The animal study was reviewed and approved by Regierungspräsidium Darmstadt, Dezernat V 54.

## Author contributions

TJ, SS, ME, and TD contributed to conception and design of the study. TJ conducted the experiments, image acquisition, and data analysis. AB and PJ performed the compartmental modeling. All authors wrote the sections of the manuscript, contributed to manuscript revision, read, and approved the submitted version.
